# Association of whole blood multi-micronutrients with mild cognitive impairment in Chinese older adults: a matched case–control study

**DOI:** 10.1007/s00394-025-03629-6

**Published:** 2025-03-06

**Authors:** Zehao Wang, Cheng Cheng, Huilian Duan, Xukun Chen, Wen Li, Fei Ma, Zhenshu Li, Jing Yan, Ruikun He, Zhongxia Li, Mengtong Yang, Zhenghua Huang, Yongjie Chen, Guowei Huang

**Affiliations:** 1 Department of Nutrition and Food Science, School of Public Health, Tianjin Medical University, Tianjin, 300070 China; 2 Tianjin Key Laboratory of Environment, Nutrition and Public Health, Tianjin, 300070 China; 3 Key Laboratory of Prevention and Control of Major Diseases in the Population, Ministry of Education, School of Public Health, Tianjin Medical University, Tianjin, China; 4 Department of Epidemiology & Biostatistics, School of Public Health, Tianjin Medical University, Tianjin, 300070 China; 5 Department of Social Medicine and Health Administration, School of Public Health, Tianjin Medical University, Tianjin, 300070 China; 6 BYHEALTH Institute of Nutrition & Health, No. 916 Huangpu Avenue East, Yuzhu Sub-district, Huangpu District, Guangzhou, 510700 China; 7 Department of Critical Care Medicine and Anesthesiology, Tianjin Medical University General Hospital, Tianjin, 300052 China; 8 The Province and Ministry Co-Sponsored Collaborative Innovation Center for Medical Epigenetics, Tianjin, China

**Keywords:** Multi-micronutrients, Mild cognitive impairment, Matched case–control study, Older adults, Dried blood spot, Prediction model

## Abstract

**Purpose:**

Adequate micronutrients play a crucial role in cognitive health. Identifying relevant micronutrients and constructing risk prediction models can guide the prevention of mild cognitive impairment (MCI) in older adults. This study aimed to assess the associations of MCI with whole blood micronutrient levels and develop a nomogram for personalized MCI risk prediction in older adults.

**Methods:**

In the matched case–control study, 100 MCI patients and 100 matched controls by age, sex and education from Baodi District, Tianjin, China were recruited. MCI was determined by a modified version of the Petersen criteria. Whole blood levels of 9 vitamins and 5 minerals were measured using the dried blood spot technique. Weighted quantile sum regression was employed to identify the most significant micronutrients associated with cognitive function. Receiver operating characteristic (ROC) curves were constructed, and a nomogram for predicting MCI risk was developed.

**Results:**

High levels of vitamins (vitamin A, vitamin B_2_, vitamin B_6_, vitamin B_9_) and minerals (magnesium, selenium) were significantly associated with lower MCI prevalence, in which vitamin B_2_, vitamin B_9_ and selenium were ranked as the most significant contributors to cognitive function. The ROC curves for vitamin B_2_ and vitamin B_9_ (area under the curve = 0.855) have superior diagnostic accuracy compared to individual assessments (*p* < 0.05). Based on these findings, a nomogram was developed using these two micronutrients to predict MCI risk.

**Conclusion:**

The nomogram based on vitamin B_2_ and vitamin B_9_ can be effectively used to detect MCI early and guide preventive strategies in older adults.

## Introduction

With global increases in life expectancy and population, the number of aging individuals is rising, leading to a growing prevalence of age-related neurodegenerative conditions, including dementia [1]. Alzheimer's disease (AD), the most common form of irreversible dementia, places a significant burden on both patients and society [2]. In the absence of effective therapies for AD, mild cognitive impairment (MCI) represents a critical transitional phase between healthy aging and dementia, offering a window for interventions that may slow the progression to dementia [3]. Given that many MCI risk factors, such as diabetes, physical inactivity and social isolation are modifiable, prevention strategies are crucial [4]. Specifically, mounting evidence supports the importance of favorable nutrition for cognitive function and its association with reduced risk of MCI [5].

Micronutrients, including vitamins and minerals, have been implicated in the pathogenesis of certain neurological disorders due to prolonged abnormal levels, which may contribute to severe complications in neurological diseases [6]. Currently, widespread micronutrient deficiencies among the older population negatively impact the health of many older adults [7]. Emerging evidence suggests that micronutrients may influence key pathways or pathological processes related to MCI, such as DNA repair, mitochondrial function, and gene networks involved in protein encoding [8, 9]. Notably, previous randomized clinical trials have shown that daily multivitamin-mineral supplementation can significantly improve overall cognition compared to placebo [10, 11].

Previous studies have explored the relationship between multiple micronutrients and MCI. For example, Mustafa et al. summarized and published a scoping review on the relationship between micronutrient malnutrition and mild cognitive impairment in the elderly, showing that widespread micronutrient deficiencies are associated with mild cognitive impairment [12]. Li R et al. examined the association between nutrient intake with pro-inflammatory or anti-inflammatory potential and the risk of MCI in older adults in northern China by assessing the intake of 22 nutrients. However, the study relied on self-reported dietary data and did not develop a specific risk prediction model [13]. The dried blood spot (DBS) technique, which requires only 20–40 μL of blood from venous or fingertip samples, offers significant advantages for micronutrient detection [14]. DBS samples are easy to collect, store, and transport, making them particularly suitable for older populations [15, 16]. Despite these advantages, there is limited research on developing predictive models for MCI based on micronutrient levels in older Chinese adults, especially using the DBS technique for precise quantification.

In this context, this matched case–control study aimed to investigate the relationship of multiple micronutrients with MCI, then rank and quantify the most significant micronutrients, and develop a nomogram for individualized MCI risk prediction in older adults.

## Methods

### Study participants

**Fig. 1:**
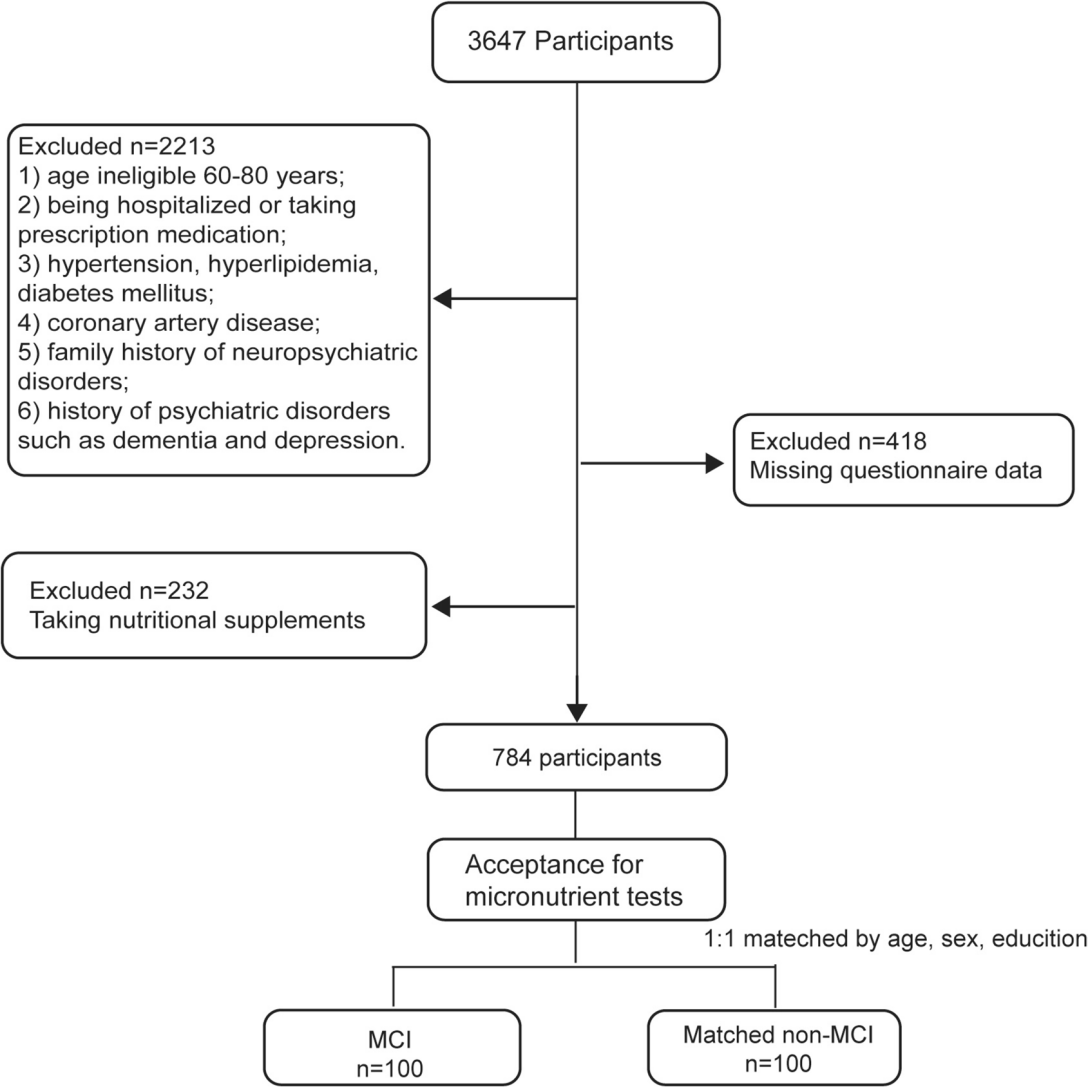
Flow chart with details of study sample derivation. *MCI* mild cognitive impairment

Participants for this study were recruited from Baodi District, Tianjin, China. The population in this study originated from the Tianjin Elderly Nutrition and Cognition cohort study (Clinical Trials Registration Identifier: ChiCTR2000034348). The total number of the cohort study was 7304, among which 3647 individuals consented to participate in this study and had their blood samples collected. The exclusion criteria were as follows: age outside the 60–80 years range, being hospitalized or taking prescription medication, having chronic conditions such as hypertension, hyperlipidemia, or diabetes mellitus, suffering from coronary artery disease, having a family history of neuropsychiatric disorders, or diagnosed with psychiatric disorders, including dementia, depression, and sleep disorders. For each case of MCI, a matched non-MCI control was randomly selected from the community. Propensity score matching was used to match cases and controls based on age (± 1 year), sex and education level (± 1 level). Based on previous studies, the parameters used for the sample size calculation in this study were OR = 3.07, *p*_0_ = 0.15, *α* = 0.05, *β* = 0.2 [17]. Using the 1:1 matched case–control study design, a minimum of 95 individuals per group was required. This study recruited 100 participants in each group to meet the sample size requirement. The protocol was approved by the Ethics Committee of Tianjin Medical University, China (Approval No. TMUhMEC2018013) and the Ethics Committee of Baodi Clinical College of Tianjin Medical University, China (Approval No. BDYYLL202404). All participants provided written informed consent, and for illiterate participants, informed consent was given by their legal representatives. The study followed the principles of the Declaration of Helsinki. A flowchart of the recruitment process is shown in Fig. 1.

### Assessment of MCI

The detection of MCI currently relies on the Petersen criteria diagnostic guidelines, which have been pivotal in distinguishing MCI from normal cognitive aging and dementia, particularly Alzheimer's disease [18]. In this study, MCI was diagnosed using a modified version of the Petersen criteria, which included the following: (1) at least six months of subjective memory complaints; (2) a Mini-Mental State Examination (MMSE) scored of ≤ 17 points for illiterate subjects, ≤ 20 points for primary education, and ≤ 24 points for secondary education and above; (3) no diagnosis of dementia (according to the criteria of the Diagnostic and Statistical Manual of Mental Disorders, Fourth Edition), Alzheimer's disease (AD) (according to the criteria of the National Institute of Neurological and Communicative Disorders and Stroke and the Alzheimer's Disease and Related Disorders Association), psychiatric disorders, brain injury, or physical illnesses resulting in cognitive deficits; (4) cognitive performance on at least one neuropsychological test below 1.5 SDs from the age-corrected norm in the neuropsychological battery; (5) no or mild impairment in activities of daily living, as indicated by a score of < 26 points on the Activities of Daily Living (ADL) scale, which is a convenient assessment tool that provides self-reported information on the functional skills required for daily living [19]; Patients with MCI must meet all criteria. The diagnosis of MCI was confirmed by a consensus panel of physicians, neurologists, and medical specialists.

### Data collection

Demographic characteristics and lifestyle factors were collected through structured questionnaires by trained interviewers. Anthropometric measurements, including weight (kg), height (m), and waist circumference (WC, cm), were taken by trained staff. Body mass index (BMI) was calculated as weight divided by squared height (kg/m^2^). Education level was categorized into three strata: “Illiteracy”, “Elementary school”, “Secondary school and higher”. Marital status was categorized as “Married and cohabited” or “Single, separated or widowed”. Smoking status was categorized as “Smoker” and “Non-smoker and ex-smoker”, while drinking status was categorized as “Drinker” and “Non-drinker and ex-drinker”.

### Laboratory methods of dried blood spot (DBS)

#### Whole blood collection

Whole blood samples (5 mL) were collected from each participant via venipuncture, stored in EDTA vacutainer tubes, and kept at – 80 °C until analysis. The collection was performed by hospital nursing staff.

#### Chemicals and reagents for DBS

Deionized water (Milli-Q IQ 7000 water purification system, Merck & Co., Inc., Rahway, N.J., USA) was used throughout the experiment. Phosphate buffered saline (PBS) was purchased from Thermo Fisher Scientific Inc. (Waltham, MA, USA). Nitric acid and Triton x-100, used for blood treatment, were of superior purity (99.8%). The helium used in the instrument was of ≥ 99.995% purity. Cysteine hydrochloride for liquid extract was of ≥ 99%. Vitamin standards and bovine serum albumin (BSA) were purchased from Sigma-Aldrich, Methanol (LC–MS grade), 2-propanol (HPLC grade), acetonitrile (HPLC grade), and formic acid (LC–MS grade) were purchased from Thermo Fisher Scientific Inc. DBS cards were purchased from CAMAG, Germany.

#### Preparation of experimental solutions and QC samples

A surrogate blood matrix was prepared by adding 2% bovine serum albumin (BSA) in PBS and stored at – 20 C until use. Vitamin solution (50 μg/mL) and internal standard (IS) solution (40 μg/mL) were prepared and stored at – 20 °C. The standard curve working solution was prepared by diluting the vitamin solution and the blood substitute matrix to the final concentrations of 2, 5, 10, 20, 40, 80 and 120 ng/mL. The QC working solution was prepared similarly, with final concentrations of 2, 5, 50 and 100 ng/mL. For mineral detection, a mixed standard working solution was prepared by diluting the original solution (GNM-M104332-2013, Guobiao Testing & Certification Co., Ltd.) in an extraction solution (0.5% nitric acid + 1 g/L cysteine + 0.01% Triton X-100). The quality of each element in the standard solution series was determined according to instrument instruction (The Agilent 7800 Inductively Coupled Plasma Mass Spectrometer, G8421A, Santa Clara, CA, USA). Customized mixed standard solutions were prepared to detect minerals such as iron, magnesium, zinc (100 mg/L), copper and selenium (1 mg/L). The internal standard element reserve solution (100 mg/L) was diluted to 100 ng/mL with deionized water.

#### Preparation and detection of DBS sample

Micronutrients including vitamin A, vitamin D, vitamin E, vitamin B_1_, vitamin B_2_, vitamin B_3_, vitamin B_5_, vitamin B_6_, vitamin B_9_, magnesium, copper, iron, zinc and selenium were examined in this study. Vitamin B_12_ levels were not assessed directly due to assay instability; instead, methylmalonic acid (MMA) was used as a proxy marker. A 20 μL sample of whole blood was pipetted onto the center of a DBS cassette, dried for 3 h in a sealed foil pouch away from light, and stored at −80 °C until analysis. Before analysis, samples were loaded into the DBS MS500 Sample Rack. The cassette was transferred to the IS Spray Module and sprayed with 10 μL IS solution over 10 s. After drying for 30 s, the sample was transferred to the extraction platform, where 15 μL of extract (methanol: water = 7:3) was extracted at a flow rate of 50 μL per minute. The eluate (15 μL) was loaded into the sample ring (10 μL) and injected into the integrated LC–MS (Sciex Triple Quad 6500 mass spectrometer) and ICP-MS (7800 Agilent Inductively Coupled Plasma Mass Spectrometry) instrument (full ring injection) for vitamin and mineral analysis. The extraction platform was then rinsed with extraction buffer, rinse solution (methanol: water: acetonitrile: 2-propanol = 1: 1: 1: 1) and extraction buffer for 30 s each.

### Statistical analysis

Participant characteristics are presented as the mean (SD) for continuous variables, and counts (percentages) for categorical variables. Depending on normality, paired t-tests or Wilcoxon signed-rank sum tests were used for continuous variables and McNemar’s test was used for categorical variables to compare cases and controls. In this study, Model 1 was unadjusted, while Model 2 adjusted for marriage, smoking status, alcohol drinking, BMI and WC. Conditional logistic regression models were used to test the association between related factors and MCI occurrence, estimating each micronutrient's odds ratio (OR) and 95% confidence interval (*CI*). Weighted quantile sum (WQS) regression, a common multiple regression model for high-dimensional data sets, was employed to assess the contribution of different micronutrients to cognitive function. The WQS index’s relationship with the MMSE score was analyzed, and micronutrients with high *β* coefficients in WQS were integrated into a receiver operating characteristic (ROC) analysis to evaluate sensitivity and specificity for detecting MCI at various thresholds. The area under the curve (AUC) was used to determine predictive value, with AUC > 0.7 considered excellent. The diagnostic accuracy of different micronutrient metrics is compared by evaluating AUC values. A nomogram was then created to predict MCI risk based on micronutrient levels. Statistical significance was set at *p* < 0.05 for all hypotheses. All analyses were performed using SPSS 25.0 (IBM Corp., Armonk, NY, USA) and R software version 4.3.2 (R Core Team).

## Results

### Demographical and clinical features

The demographic and clinical characteristics of the case–control pairs are shown in Table 1. In this study, 100 (49 male and 51 female) eligible cases of MCI and 100 healthy controls were included. Both groups had a median age of 72 years. Compared to controls, MCI cases exhibited significantly lower WC, MMSE scores and higher ADL scores (*p* < 0.05). No significant differences were observed between cases and controls regarding age, sex, education levels, smoking status, alcohol drinking, BMI and homocysteine (Hcy) (*p* > 0.05).

**Table 1:** Descriptive characteristics of the study population

Characteristics	MCI (n = 100)	Non-MCI (n = 100)	*P* value
Age (years)	71.05 ± 5.18	71.11 ± 5.04	0.333
Sex, male (%)	49 (49)	49 (49)	1
Education levels, n (%)			0.129
Illiteracy	20 (20)	23 (23)	
Primary school	26 (26)	36 (36)	
Secondary and higher	54 (54)	41 (41)	
Marital status, n (%)			0.175
Married and cohabited	86 (86)	92 (92)	
Single, separated or widowed	14 (14)	8 (8)	
Smoking status, n (%)			0.653
Smoker	35 (35)	32 (32)	
Non-smoker and ex-smoker	65 (65)	68 (68)	
Alcohol drinking, n (%)			0.868
Drinker	24 (24)	23 (23)	
Non-drinker and ex-drinker	76 (76)	77 (77)	
BMI (kg/m^2^)	19.35 ± 4.27	25.61 ± 3.30	0.075
WC (cm)	86.52 ± 7.95	90.71 ± 7.86	< 0.001
MMSE*	24.79 ± 2.94	24.83 ± 3.09	< 0.001
ADL*	16.92 ± 5.69	15.11 ± 3.41	< 0.001
Hcy* (μmol/L)	14.32 ± 7.81	14.32 ± 3.76	0.089

Values are mean ± SD

*MCI* mild cognitive impairment; *BMI* body mass index; *WC* waist circumference; *MMSE* minimum mental state examination; *ADL* activity of daily living; *Hcy* homocysteine

^*^Means the indicators do not follow the normal distribution

### Comparison of the micronutrient levels between the cases and controls

The levels of micronutrients, including vitamin A, vitamin D, vitamin E, vitamin B_1_, vitamin B_2_, vitamin B_3_, vitamin B_5_, vitamin B_6_, vitamin B_9_, magnesium, copper, iron, zinc and selenium, are shown in Table 2. Vitamin B_12_ levels were not assessed directly due to assay instability; instead, MMA was used as a proxy marker. The results indicated that controls had significantly higher levels of vitamin A, vitamin B_2_, vitamin B_6_, vitamin B_9_, as well as magnesium, iron, and selenium compared to MCI cases (*p* < 0.05).

**Table 2:** Comparison of the whole blood micronutrient levels between the cases and controls

Micronutrients	MCI (n = 100)	Non-MCI (n = 100)	*P* value
Vitamin A (ng/mL)	279.54 ± 104.85	319.39 ± 111.93	0.004
Vitamin D (ng/mL)	31.54 ± 8.25	33.71 ± 12.43	0.135
Vitamin E (μg/mL)	3.22 ± 1.38	3.44 ± 1.10	0.457
Vitamin B_1_ (ng/mL)	0.79 ± 0.56	0.88 ± 0.59	0.130
Vitamin B_2_ (ng/mL)	2.63 ± 1.94	4.54 ± 2.77	< 0.001
Vitamin B_3_ (μg/mL)	9.13 ± 2.19	9.74 ± 2.89	0.190
Vitamin B_5_ (μg/mL)	68.98 ± 18.24	74.19 ± 49.72	0.918
Vitamin B_6_ (ng/mL)	9.05 ± 3.06	10.29 ± 3.86	0.034
Vitamin B_9_ (ng/mL)	2.83 ± 1.29	4.83 ± 2.05	< 0.001
MMA* (nmol/L)	249.21 ± 121.91	218.59 ± 87.79	0.182
Magnesium (μg/mL)	36.18 ± 5.92	38.77 ± 6.67	0.008
Copper (μg/mL)	0.85 ± 0.20	0.84 ± 0.16	0.755
Iron (μg/mL)	381.27 ± 68.67	400.57 ± 68.86	0.026
Zinc (μg/mL)	7.12 ± 3.05	7.50 ± 2.36	0.063
Selenium (ng/mL)	92.51 ± 23.39	114.53 ± 25.90	< 0.001

The variables did not conform to normal distribution and Wilcoxon signed rank sum test was used

*MMA* methylmalonic acid; *MCI* mild cognitive impairment

^*^Considering the instability of vitamin B_12_ in the assay and the higher sensitivity and specificity of MMA in detecting vitamin B_12_ deficiency, MMA levels were used instead of vitamin B_12_ in this study

### Associations between micronutrient levels and MCI

**Fig. 2:**
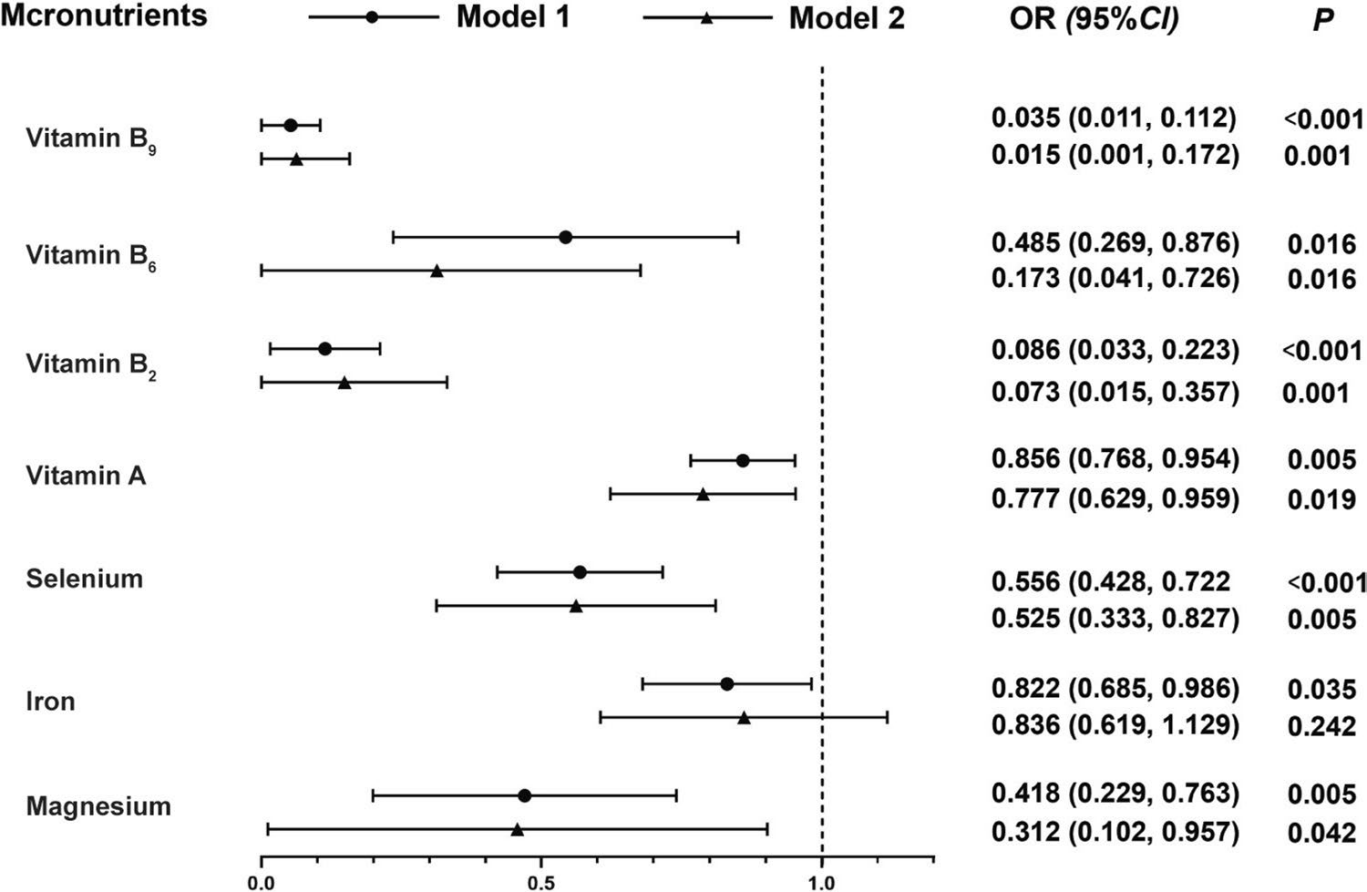
The conditional logistic regression on micronutrient levels related to MCI. Data were square-root transformed according to data characteristics. Model 1 is the non-adjusted model; Model 2 is adjusted for marriage, smoking status, alcohol drinking, BMI and WC. *MCI* mild cognitive impairment; *BMI* body mass index; *WC* waist circumference; *OR* odds ratio

Conditional logistic regression model was used to assess the association between micronutrient levels and MCI, as illustrated in Fig. 2. Data were square-root transformed according to data characteristics. The results of Model 2 showed that the higher levels of vitamin A (OR = 0.777, 95% *CI* = 0.629–0.959), vitamin B_2_ (OR = 0.073, 95% *CI* = 0.015–0.357), vitamin B_6_ (OR = 0.173, 95% *CI* = 0.041–0.726), vitamin B_9_ (OR = 0.015, 95% *CI* = 0.001–0.172), magnesium (OR = 0.312, 95% *CI* = 0.102–0.957) and selenium (OR = 0.525, 95% *CI* = 0.333–0.827) were significantly associated with lower MCI prevalence, while other micronutrient levels had no significant associations with MCI prevalence.

### Weighted quantile sum (WQS) regression analysis

**Fig. 3:**
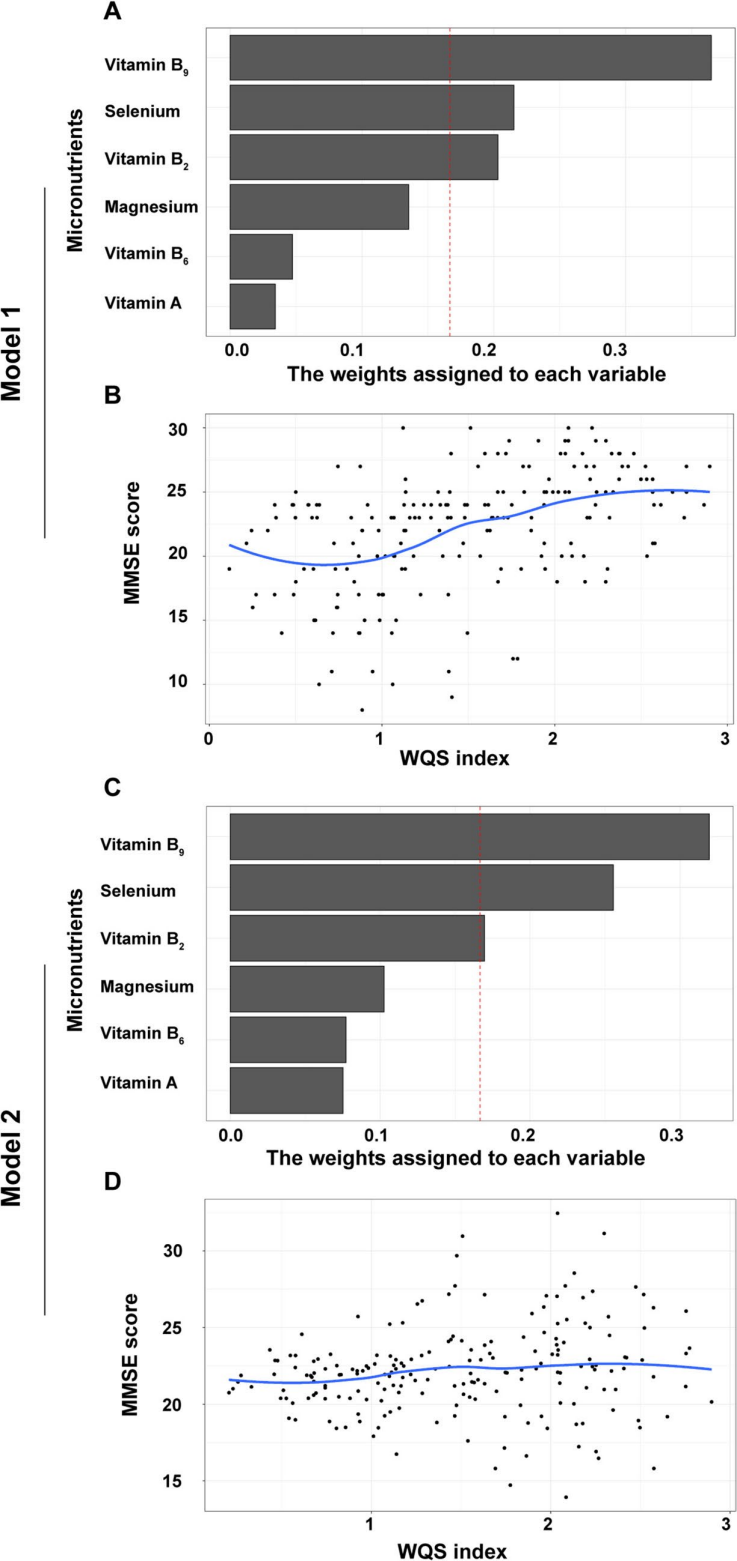
The WQS regression of micronutrients and MMSE score. Model 1 is non-adjusted; Model 2 is adjusted for age, education, smoking status, alcohol drinking, BMI and WC. **A** The weights assigned to each micronutrients in Model 1. **B** Correlation between WQS index and MMSE scores in Model 1. **C** The weights assigned to each micronutrients in Model 2. **D** Correlation between WQS index and MMSE scores in Model 2. *WQS* weighted quantile sum; *MMSE* Mini-Mental State Examination; *BMI* body mass index; *WC* waist circumference

The weight index estimated by WQS regression indicates the relative importance of each micronutrient in MMSE scores. The results showed that after adjusting for marriage, smoking status, alcohol drinking, BMI and WC, vitamin B_9_ (*β* = 0.320, 95% *CI* = 0.083–0.491), selenium (*β* = 0.256, 95% *CI* = 0.171–0.416), vitamin B_2_ (*β* = 0.170, 95% *CI* = 0.029–0.306) had strong associations with MMSE score. The positive correlation between the WQS index and MMSE scores suggests that higher levels of these micronutrients are linked to better cognitive function (Fig. 3). The detailed results of the non-adjusted Model 1 and the adjusted Model 2 are shown in Table 3.

**Table 3:** Association between WQS index and MMSE score

Outcomes	Model 1		Model 2	
	β	95% CI	β	95% CI
Vitamin B_9_	0.365	(0.230, 0.436)	0.320	(0.083, 0.491)
Selenium	0.215	(0.163, 0.285)	0.256	(0.171, 0.416)
Vitamin B_2_	0.203	(0.074, 0.374)	0.170	(0.029, 0.306)
Magnesium	0.135	(0.074, 0.195)	0.103	(0.066, 0.141)
Vitamin B_6_	0.047	(0.003, 0.109)	0.077	(0.015, 0.146)
Vitamin A	0.034	(0.005, 0.059)	0.075	(0.005, 0.206)

Model 1 is non-adjusted; Model 2 is adjusted for age, education, smoking status, alcohol drinking, body mass index and waist circumference. *WQS* weighted quantile sum; *MMSE* minimum mental state examination

### Receiver operating curve (ROC) analysis

**Fig. 4:**
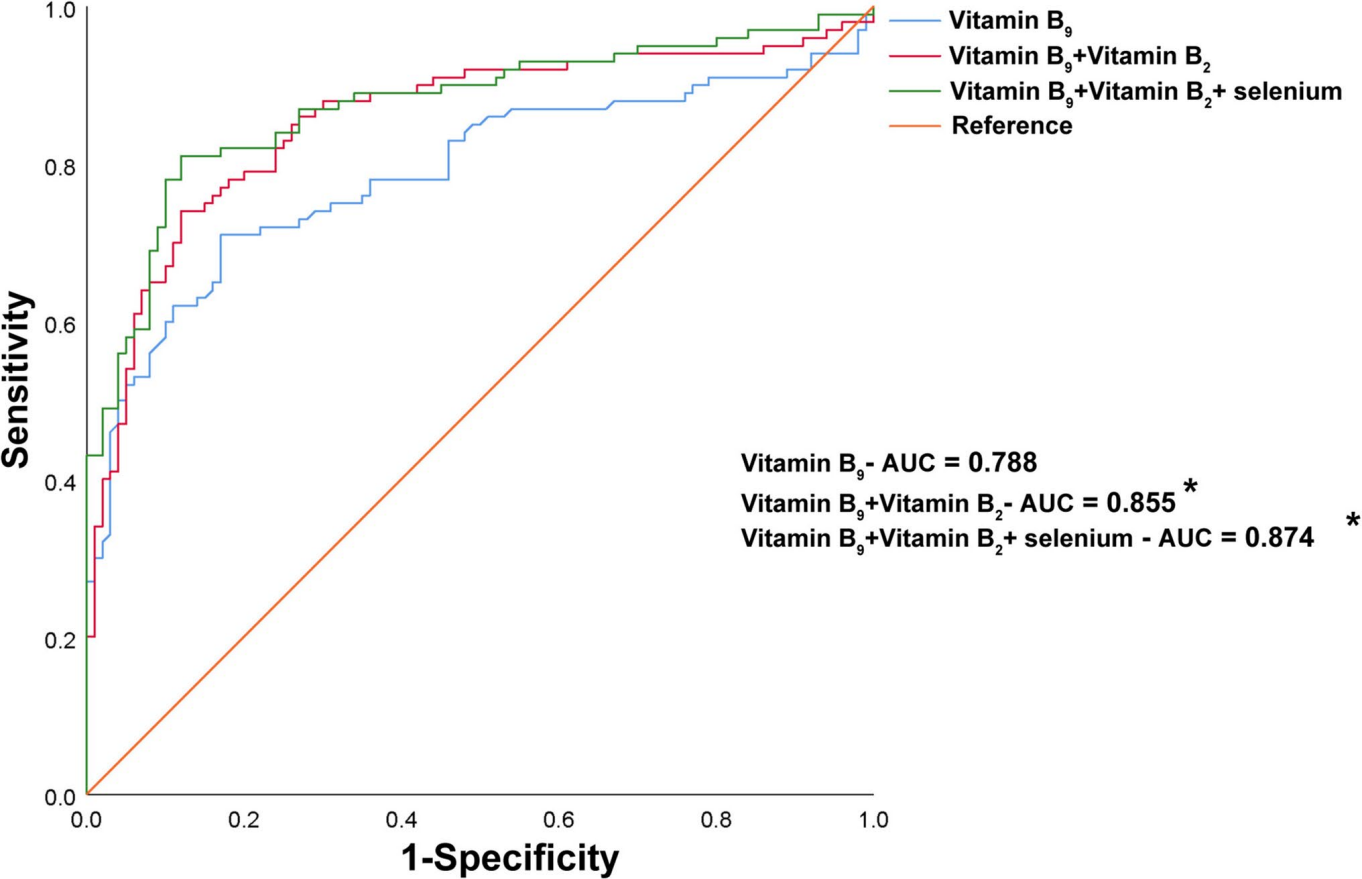
ROC curves to evaluate the utility of levels of micronutrients for the discrimination of MCI patients. *Indicates the AUC values significantly different from vitamin B_9_. *ROC* receiver operating characteristic; *MCI* mild cognitive impairment; *AUC* area under the curve

Micronutrients identified through WQS regression (vitamin B_2_, vitamin B_9_ and selenium) were analyzed in the ROC model to assess their diagnostic value. The ROC curves in Fig. 4 showed that vitamin B_9_ alone had the highest area under the curve (AUC) at 0.788. The combination of vitamin B_9_ and vitamin B_2_ yielded an AUC of 0.855, and the inclusion of all three micronutrients (vitamin B_2_, vitamin B_9_ and selenium) resulted in an AUC of 0.874. The combination of vitamins B_2_ and vitamin B_9_ was the most effective for diagnosing MCI, as its AUC was comparable to the combination of all three micronutrients and significantly better than other combinations. Supplementary Material provides AUC values for additional individual micronutrients and combinations.

### Nomogram analysis

**Fig. 5:**
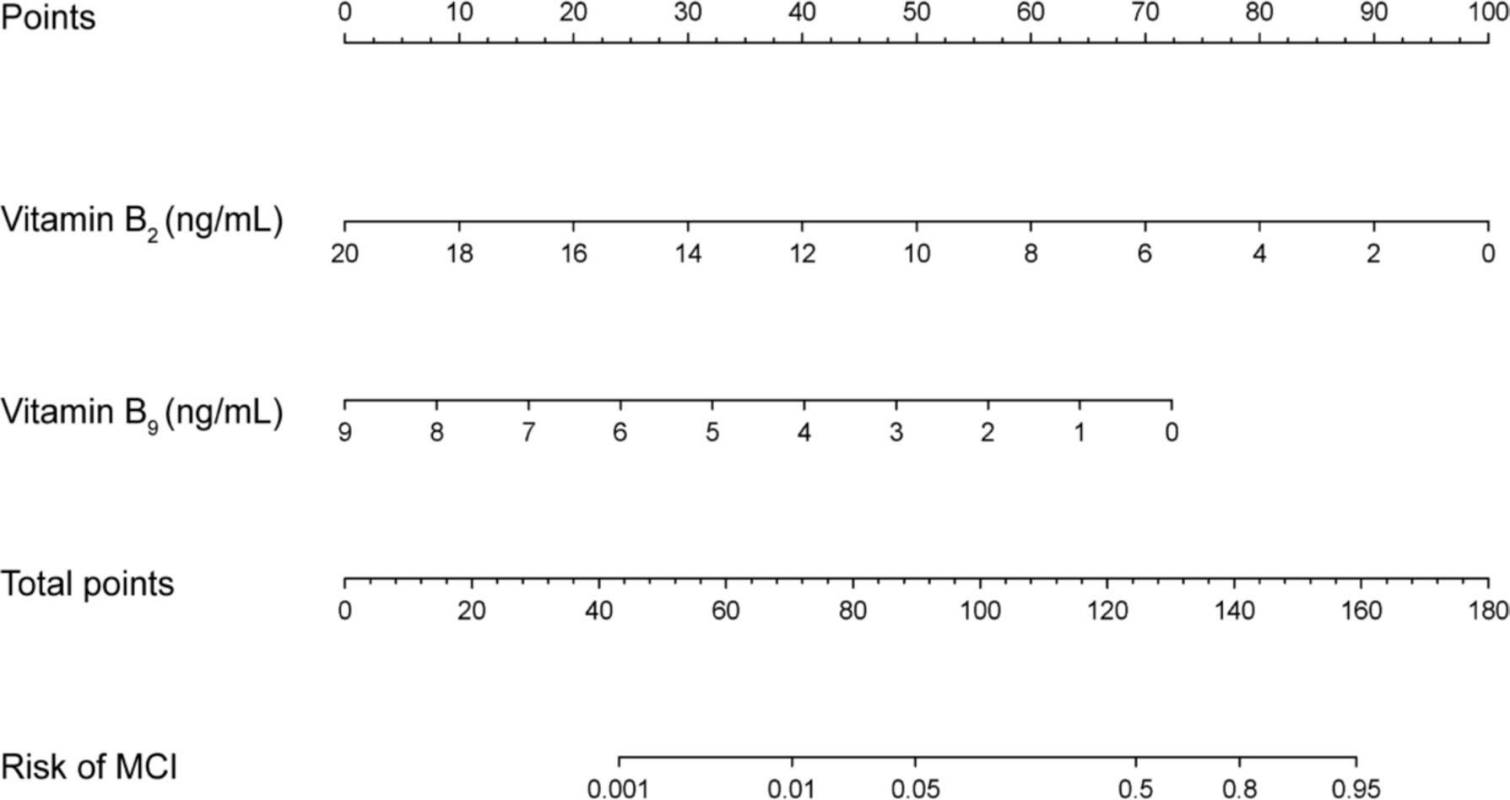
Nomogram analysis of vitamin B_2_ and vitamin B_9_ with risk of MCI. *MCI* mild cognitive impairment

A nomogram incorporating vitamin B_2_ and vitamin B_9_ was developed to represent the risk prediction model for MCI visually. The nomogram assigns scores to each micronutrient, with the total score corresponding to the predicted MCI risk. The model demonstrated excellent discrimination with a concordance index (C-index) of 0.855, indicating great predictive capability (Fig. 5).

## Discussion

This study revealed the significant association between micronutrients (vitamin A, vitamin B_2_, vitamin B_6_, vitamin B_9_, magnesium, selenium) and the risk of MCI. Through the screening process using WQS regression and ROC analysis, we identified a combination of vitamin B_2_ and vitamin B_9_ as particularly effective in predicting MCI occurrence. Then, we developed a nomogram based on whole blood levels of vitamin B_2_ and vitamin B_9_ to construct a predictive model for MCI. Based on our retrieval situation, this is the first study to utilize an accessible DBS technique to explore the association between micronutrients and MCI in Chinese older adults.

The results linking MCI to multiple blood levels of vitamins are consistent with those of previous studies [8, 20]. In this study, higher levels of vitamin A, vitamin B_2_, vitamin B_6_ and vitamin B_9_ were related to a lower MCI prevalence, even after adjusting for potential confounders. These results align with earlier studies demonstrating that elevated levels of these vitamins can significantly enhance cognitive function [21–23]. For instance, vitamin A deficiency has been linked to impaired retinoic acid signaling, which may contribute to normal age-related cognitive decline and Alzheimer’s disease [24]. Similarly, vitamin B_6_ deficiency has been shown to impair cognitive function through its effects on noradrenergic signaling, as observed in animal models [25]. Vitamin B_2_, known as riboflavin, is crucial in several neurodegenerative pathways, including antioxidant activity, mitochondrial function, iron metabolism, and myelination [26]. Vitamin B_9_ is essential for neurological activity, particularly in producing and maintaining new cells, synthesizing nucleotides, and regulating methyl group availability in the brain [27].

In terms of minerals, our study found a significant association between magnesium, selenium and MCI, which is consistent with previous studies. Minerals may be linked to brain health through various biological pathways. Higher magnesium levels in the brain have been shown to reduce oxidative stress, systemic inflammation, and neurodegeneration while enhancing synaptic plasticity [28]. Selenium is vital for brain health, with selenoprotein P offering neuroprotective effects by preventing oxidative damage and supporting cognitive function [29]. Evidence from human studies supports the role of Se in mitigating cognitive decline, as lower plasma Se concentrations have been linked to reduced cognitive performance [30, 31].

The detection of various micronutrients in whole blood using DBS technology can minimize the inaccuracies from the individual testing of single components in the mixture. After adjusting for covariates, WQS analysis ranked vitamin B_2_, vitamin B_9_ and selenium as the most significant contributors to cognitive function. The positive correlation between the WQS index and MMSE scores further suggests that higher levels of these micronutrients are associated with better cognitive function. While vitamin A and vitamin B_6_ also showed associations with lower MCI prevalence in univariate analyses, their contributions to cognitive function appeared less pronounced when evaluated in the context of the whole micronutrient mixture. Nonetheless, it is essential to consider the potential interactions between these micronutrients, as factors with fewer individual effects may still play a meaningful role in the overall mixture.

This study has several strengths. First, we utilized the DBS technique for blood sample collection, which requires only a small amount of blood and is particularly advantageous for remote or mobility-limited populations [32]. Second, using WQS regression allowed for a holistic assessment of micronutrient contributions, considering correlations between exposures and outcomes. Finally, by focusing on the most relevant micronutrients (vitamin B_2_ and vitamin B_9_), we developed a highly effective risk prediction model for MCI.

Our study also has limitations that need to be considered. First, as a matched case–control study, we cannot confirm the causality of the finding. Second, the risk prediction model also requires external validation in independent cohorts. Third, the WQS regression assumes linear and cumulative effects of individual exposures, which may not fully capture the complexity of real-world epidemiologic data [33]. Finally, whether there is any link between the micronutrients regarding physiological and biochemical mechanisms requires further molecular-level studies.

In conclusion, we examined the levels of 14 micronutrients and MMA, which is the proxy marker of vitamin B_12_ in the whole blood of older adults with and without MCI using the DBS technique. Through logistic regression and WQS regression, we identified vitamin B_2_, vitamin B_9_ and selenium as the micronutrients most closely related to cognitive function. The ROC analysis confirmed that the combination of vitamin B_2_ and vitamin B_9_ provides an excellent means of predicting MCI prevalence, and we developed a corresponding nomogram. This study lays the groundwork for protecting cognitive function and predicting MCI risk in older adults from a micronutrient perspective.

## Data Availability

The raw data supporting the conclusions of this article will be available upon request from the corresponding author.
